# Frequency of positive antiphospholipid antibodies in pregnant women with SARS-CoV-2 infection and impact on pregnancy outcome: A single-center prospective study on 151 pregnancies

**DOI:** 10.3389/fimmu.2022.953043

**Published:** 2022-09-15

**Authors:** Giorgia Ingrid Gozzoli, Elda Piovani, Beatrice Negri, Margaret Mascherpa, Rossana Orabona, Cristina Zanardini, Sonia Zatti, Silvia Piantoni, Maria Grazia Lazzaroni, Cesare Tomasi, Federico Prefumo, Enrico Sartori, Franco Franceschini, Angela Tincani, Laura Andreoli

**Affiliations:** ^1^ Rheumatology and Clinical Immunology Unit, Azienda Socio Sanitaria Territoriale (ASST) Spedali Civili, Brescia, Italy; ^2^ Department of Clinical and Experimental Sciences, University of Brescia, Brescia, Italy; ^3^ Division of Obstetrics and Gynaecology, ASST Spedali Civili, Brescia, Italy

**Keywords:** anti-phospholipid antibodies, anti-phospholipid syndrome, anti-beta2glycoprotein I antibodies, COVID-19, SARS-CoV-2, pregnancy morbidity, HELLP syndrome, preeclampsia

## Abstract

**Background:**

At the beginning of the SARS-CoV-2 pandemic, there was a lack of information about the infection’s impact on pregnancy and capability to induce *de novo* autoantibodies. It soon became clear that thrombosis was a manifestation of COVID-19, therefore the possible contribution of *de novo* antiphospholipid antibodies (aPL) raised research interest. We aimed at screening SARS-CoV-2 positive pregnant patients for aPL.

**Methods:**

The study included consecutive pregnant women who were hospitalized in our Obstetric Department between March 2020 and July 2021 for either a symptomatic SARS-CoV-2 infection or for other reasons (obstetric complications, labour, delivery) and found positive at the admission nasopharyngeal swab. All these women underwent the search for aPL by means of Lupus Anticoagulant (LA), IgG/IgM anti-cardiolipin (aCL), IgG/IgM anti-beta2glycoprotein I (aB2GPI). Data about comorbidities, obstetric and neonatal complications were collected.

**Results:**

151 women were included. Sixteen (11%) were positive for aPL, mostly at low titre. Pneumonia was diagnosed in 20 women (5 with positive aPL) and 5 required ICU admission (2 with positive aPL). Obstetric complications occurred in 10/16 (63%) aPL positive and in 36/135 (27%) negative patients. The occurrence of HELLP syndrome and preeclampsia was significantly associated with positive aPL (p=0,004). One case of maternal thrombosis occurred in an aPL negative woman. aPL positivity was checked after at least 12 weeks in 7/16 women (44%): 3 had become negative; 2 were still positive (1 IgG aB2GPI + IgG aCL; 1 IgM aB2GPI); 1 remained positive for IgG aCL but became negative for aB2GPI; 1 became negative for LA but displayed a new positivity for IgG aCL at high titre.

**Conclusions:**

The frequency of positive aPL in pregnant women with SARS-CoV-2 infection was low in our cohort and similar to the one described in the general obstetric population. aPL mostly presented as single positive, low titre, transient antibodies. The rate of obstetric complications was higher in aPL positive women as compared to negative ones, particularly hypertensive disorders. Causality cannot be excluded; however, other risk factors, including a full-blown picture of COVID-19, may have elicited the pathogenic potential of aPL and contributed themselves to the development of complications.

## Introduction

The SARS-CoV-2 pandemic has been affecting healthcare systems worldwide since 2020. At the beginning, there was a lack of information about the impact of the infection on pregnancy and its capability to induce *de novo* autoantibodies. It soon became clear that thrombosis was a manifestation of COVID-19, therefore the possible contribution of *de novo* antiphospholipid antibodies (aPL) raised research interest, as these autoantibodies are directly involved in the pathogenesis of vascular and obstetric complications (1), leading to the so-called picture of antiphospholipid syndrome (APS).

Current data suggest that pregnant people with SARS-CoV-2 symptomatic infection have increased risk of caesarean section, preterm birth and fetal distress, neonatal complications, isolated hypertension, preeclampsia/eclampsia, HELLP syndrome (characterized by haemolysis, elevated liver enzyme levels, low platelets counts) (2), intensive care unit (ICU) admission and death compared to women without COVID-19 during pregnancy (3, 4). Some of the most frequent maternal complications of COVID-19, like preeclampsia and gestational hypertension, may be also related to autoimmune diseases, in particular to the presence of aPL. Obstetric APS is characterized by fetal loss, recurrent early miscarriages, intrauterine growth restriction (IUGR), severe preeclampsia or HELLP syndrome (5, 6).

Therefore, the primary objective of this study was to evaluate the presence of antiphospholipid antibodies in pregnant patients with SARS-CoV-2 infection who were admitted to our hospital. The secondary objective was to understand whether the association between aPL positivity and COVID-19 could increase the risk of obstetric and neonatal complications.

## Materials and methods

This is a single-center prospective study on consecutive SARS-CoV-2 positive pregnant women who were hospitalized to the Obstetric Department of the ASST Spedali Civili of Brescia between March 2020 and July 2021. These women were admitted either for symptomatic SARS-CoV-2 infection or for other reasons (i.e. obstetric complications, labour and delivery) and found positive at the admission nasopharyngeal swab. The study was performed according to the principles of the Declaration of Helsinki and was approved by the Local Ethics Committee (approval number NP4187). All patients gave their written informed consent.

All these women underwent search for aPL by means of Lupus Anticoagulant (LA), IgG/IgM anti-cardiolipin (aCL), IgG/IgM anti-beta2glycoprotein I (aB2GPI). IgG/IgM aCL and aB2GPI were detected by chemiluminescence immunoassay (Bioflash ^®^, INOVA Diagnostics, Werfen Group, Barcelona, Spain); LA test was performed according to the ISTH guidelines (7), using diluted Russel Viper Venom Time (dRVVT) and Silica Clotting Time (SCT) as screening tests (Werfen Group, Barcelona, Spain). Patients who tested positive for aPL were asked to recheck the tests after at least 12 weeks.

Data about symptoms related to COVID-19 were collected: dyspnea, pneumonia (clinically and radiologically documented by chest CT), fever, cough and pharyngodynia, dysgeusia or ageusia, diarrhea or vomiting. Patients with one or more of these symptoms were classified as symptomatic.

Information in pregnancy outcomes was collected: postpartum hemorrhage (PPH) (defined as losses greater than 500 ml for vaginal delivery and 1000 ml for cesarean section), need for blood transfusion, borderline blood pressure values (systolic blood pressure –SBP- between 130 and 140 mmHg and/or diastolic blood pressure –DBP- between 80 and 90 mmHg), hypertension (SBP greater than 140mmHg and/or DBP greater than 90mmHg), threatened preterm delivery (prior to 37 weeks of pregnancy), HELLP syndrome, FGR (fetal growth restriction, refers to fetus who does not achieve the expected *in utero* growth potential due to genetic or environmental factors) (8, 9), miscarriage, preterm premature rupture of membranes (pPROM), oligohydramnios (maximum vertical pocket <2 cm), thromboembolic events, preeclampsia (gestational hypertension accompanied by one or more of the following new-onset conditions at or after 20 weeks’ gestation: proteinuria or other maternal organ dysfunction like acute kidney injury, liver involvement, neurological complications, haematological complications as thrombocytopenia, uteroplacental dysfunction or stillbirth) (10), abruptio placentae (ultrasound or clinically defined), and ICU admission. Regarding newborns, we collected data on sex, gestational age, birth weight, SGA (small for gestational age; below the 10^th^ percentile of body weight), LGA (large for gestation age; above the 90^th^ percentile of body weight) (11) and the presence of any neonatal complications.

The population was divided in two groups: “antiphospholipid negative” and “antiphospholipid positive”. The latter was further divided into three subgroups according to aPL profile: “single positive”, “double positive” and “triple positive” (12).

## Statistical analysis

The database was formatted through the Microsoft-Excel^®^ software and later imported from the IBM-SPSS^®^ software ver. 28.0.1 (IBM SPSS Inc. Chicago, Illinois). The use of the Stata^®^ software ver. 17.0 (Stata Corporation, College Station, Texas) was also considered for comparisons or implementations of test output. Normality of the distributions was assessed using the Kolmogorov-Smirnov test. Categorical variables were presented as frequencies or percentages and compared with the use of the Chi-Square test and the Fisher’s exact test, as appropriate; associations of the crosstabs were verified using standardized adjusted residuals; when the matrix was 2x2 the odds ratios were calculated. Continuous variables were presented as means (in case of a normal distribution), or medians, IQR and interval (in case of a skewed distribution).

Logistic regressions were also used to calculate the associations between predictors covariates and each of the outcome variables Y. Then, the association of the dependent variables which showed significance to univariate regression on predictors covariates (one-by-one) was tested by IBM SPSS Neural Network analysis following the Multilayer Perceptron (MLP) procedure while performing a sensitivity analysis in order to compute the importance of each predictor in determining the neural network. A two-sided α level of 0.05 was considered significant for all tests.

The authors had full access to and take full responsibility for the integrity of the data.

## Results

During the enrollment period, 151 women were tested for aPL: 87 (58%) during pregnancy and 64 (42%) in the immediate postpartum period. Their mean age was 33,3 years (IQR 29,3 – 37,5) with a median gestational age at delivery of 39 weeks (IQR 32,4 – 40).

Sixteen patients (11%) tested positive for at least one assay for aPL: 12/16 (75%) were single positive and 4/16 (25%) were double positive, all of them with positive IgG and/or IgM aB2GPI. No patient had a triple positive aPL profile. Nine patients presented a LA positive test; six were positive for aCL IgG and/or IgM; five were positive for aB2GPI IgG and/or IgM. **Table 1** shows the comparison between aPL positive and aPL negative groups regarding: presence of COVID-19 symptoms and related treatment, presence of comorbidities, obstetric complications and neonatal complications.

**Table 1:** Characteristics of 151 patients with SARS-CoV-2 divided upon the positivity for antiphospholipid antibodies (aPL).

		Total patients(n=151)	aPL negative(n=135)	aPL positive(n=16)	Single Positive(n=12)	Double Positive(n=4)	LA + (n=9)	aCL + (n=6)	aB2GPI+ (n=5)
**SARS-CoV-2 symptomatic infection**		40/151 (26%)	34/135 (25%)	6/16 (36%)	5/12 (42%)	1/4 (25%)	4/9 (44%)	2/6 (33%)	1/5 (20%)
**T** **r** **e** **a** **t** **m** **e** **n** **t**	**Antiviral drugs**	13/151 (9%)	9/135 (7%)	4/16 (25%)	3/12 (25%)	1/4 (25%)	3/9 (33%)	1/6 (17%)	1/5 (20%)
	**Corticosteroids**	21/151 (14%)	16/135 (12%)	5/16 (31%)	4/12 (3%)	1/4 (25%)	3/9 (33%)	2/6 (33%)	1/5 (20%)
	**Low dose aspirin**	2/151 (1%)	2/135 (1%)	0	0	0	0	0	0
	**LMWH**	55/151 (36%)	47/135 (35%)	8/16 (50%)	6/12 (50%)	2/4 (50%)	4/9 (44%)	3/6 (50%)	3/5 (60%)
	**HCQ**	2/151 (81%)	0	2/16 (12%)	2/12 (17%)	0	1/9 (11%)	1/6 (17%)	0
**ICU** **Admission**		5/151 (3%)	3/135 (2%)	2/16 (12%)	1/12 (8%)	1/4 (25%)	1/9 (11%)	1/6 (17%)	1/5 (20%)
**Comorbidities**		41/151 (27%)	32/135 (24%)	9/16 (56%)	7/12 (58%)	2/4 (50%)	6/9 (67%)	2/6 (33%)	3/5 (60%)
**Obstetric** **Complications**		46/151 (31%)	36/135 (27%)	10/16 (62%)	6/12 (50%)	4/4 (100%)	6/9 (67%)	3/6 (50%)	5/5 (100%)
**Neonatal** **Complications**		19/151 (13%)	16/135 (12%)	3/16 (19%)	1/12 (8%)	2/4 (50%)	1/9 (11%)	2/6 (33%)	2/5 (40%)

aPL, antiphospholipid antibodies; LA, lupus anticoagulant; aCL, anti-cardiolipin; aB2GPI, anti-beta2glycoprotein I; ICU, Intensive care unit; LMWH, Low Molecular Weight Heparin; HCQ, Hydroxychloroquine.

Forty (26%) women presented with symptoms of COVID-19 at the time of admission, the mean age was 35,4 years (IQR 32,6 –39,5) with a median gestational age at delivery of 30,6 weeks (IQR 24,8 – 33,7). 6/16 (36%) aPL positive patients displayed a SARS-CoV-2 symptomatic infection: 5 with pneumonia, of which 2 required Intensive Care Unit (ICU) admission, and 1 with dyspnea. In the aPL negative group 34/135 (25%) had COVID-19 symptoms: 15 with pneumonia, of which 3 required ICU admission, and the remaining with transient dyspnea and minor symptoms (fever, cough, etc.). 111/151 (74%) were asymptomatic for SARS-CoV-2: the mean age was 32,6 years (IQR 28,8 – 36,9) with a median gestational age at delivery of 39,4 weeks (IQR 38,7 – 40,2).

41/151 (27%) women had one or more comorbidities. Nine patients with comorbidities were in the aPL positive group: autoimmune disorders (2 ANAs positivity), chronic metabolic disorders (2 hypothyroidism, 1 obesity), chronic infectious disorder (1 chronic hepatitis B), obstetric disorders (2 multiple abortions) and other comorbidities (2 behavioural disorders, 1 coagulation factor XII deficiency, 1 hypoacusis, 1 metachromatic leukodystrophy). The remaining 32 women with comorbidities were in the aPL negative group: autoimmune disorders (2 Crohn’s disease, 2 diabetes mellitus, 1 ANAs positivity, 1 anti-thyreoglobulin antibodies positivity, 1 myasthenia gravis, 1 systemic sclerosis, 1 ulcerative rectocolitis), chronic metabolic disorders (10 hypothyroidism, 4 obesity, 1 hyperparathyroidism, 1 hypovitaminosis D), neoplastic disorders (1 anamnestic ovarian cancer, 1 anamnestic papillary thyroid carcinoma), chronic infectious disorder (1 HIV infection), obstetric disorders (1 endometriosis) and other comorbidities (4 asthma, 3 chronic hypertension, 1 anemia, 1 cholecystitis, 1 cholelithiasis, 1 chronic renal failure, 1 factor V Leiden mutation, 2 hemoglobinopathies, 1 lower extremities chronic venous disease, 1 splenectomy, 1 proteinuria, 1 rheumatic mitral regurgitation).

The obstetric complications that occurred in the whole cohort were: 11 postpartum hemorrhage, 11 preeclampsia, 5 isolated hypertension, 5 IUGR, 5 SGA, 3 chorioamnionitis, 3 hepatic disease, 3 oligohydramnios, 3 urinary tract infections, 3 threat of preterm delivery, 2 abnormal cardiotocography, 2 twin pregnancy, 1 abruptio placentae, 1 cervical dystocia, 1 disseminated intravascular coagulation, 1 gestational diabetes, 1 HELLP syndrome, 1 hyperemesis, 1 iatrogenic bladder injury, 1 internal abortion, 1 LGA, 1 maternal-fetal transfusion, 1 multiple placental infarctions, 1 placenta previa major, 1 pPROM, 1 proteinuria, 1 sepsis, 1 twin pregnancy with twin-twin transfusion syndrome.

In particular, we observed 11 cases of preeclampsia, of which 4 in aPL positive patients (1 in a patient with isolated LA positivity, and 3 in the double positivity group,2 with IgG aCL and IgG aB2GPI, 1 with LA and IgG aB2GPI); 12 cases of postpartum hemorrhage, of which one occurred in a patient with isolated positive IgM aB2GPI and one in a patient with IgG aCL and IgG aB2GPI; one case of HELLP syndrome in a double positive patient (IgG aCL and IgG aB2GPI). The latter was a 39-years-old primigravida with asymptomatic SARS-CoV-2 infection who was hospitalized for an elective cesarean section. The medical history reported behavioral disorders on treatment with sertraline, trazodone, alprazolam and amisulpride. The delivery was complicated by the onset of preeclampsia, HELLP syndrome, and postpartum hemorrhage; there were no neonatal complications. After 12 weeks, aPL tests were repeated and IgG aCL remained positive while IgG aB2GPI became negative.

One or more neonatal complications occurred in 19/151 (13%) cases. Three in the aPL positive group: 2 respiratory distress syndromes, 1 admission to neonatal ICU, 1 hypoglycemia and 1 neonatal necrotizing enterocolitis. The other neonatal complications observed in the aPL negative group were: 9 admissions to neonatal ICU, 8 respiratory distress syndrome, 6 hypoglycemia, 4 hyperbilirubinemia, 1 feto-maternal transfusion, 1 sepsis, 1 transient tachypnea of the newborn, 1 urinoma.

**Figure 1:**
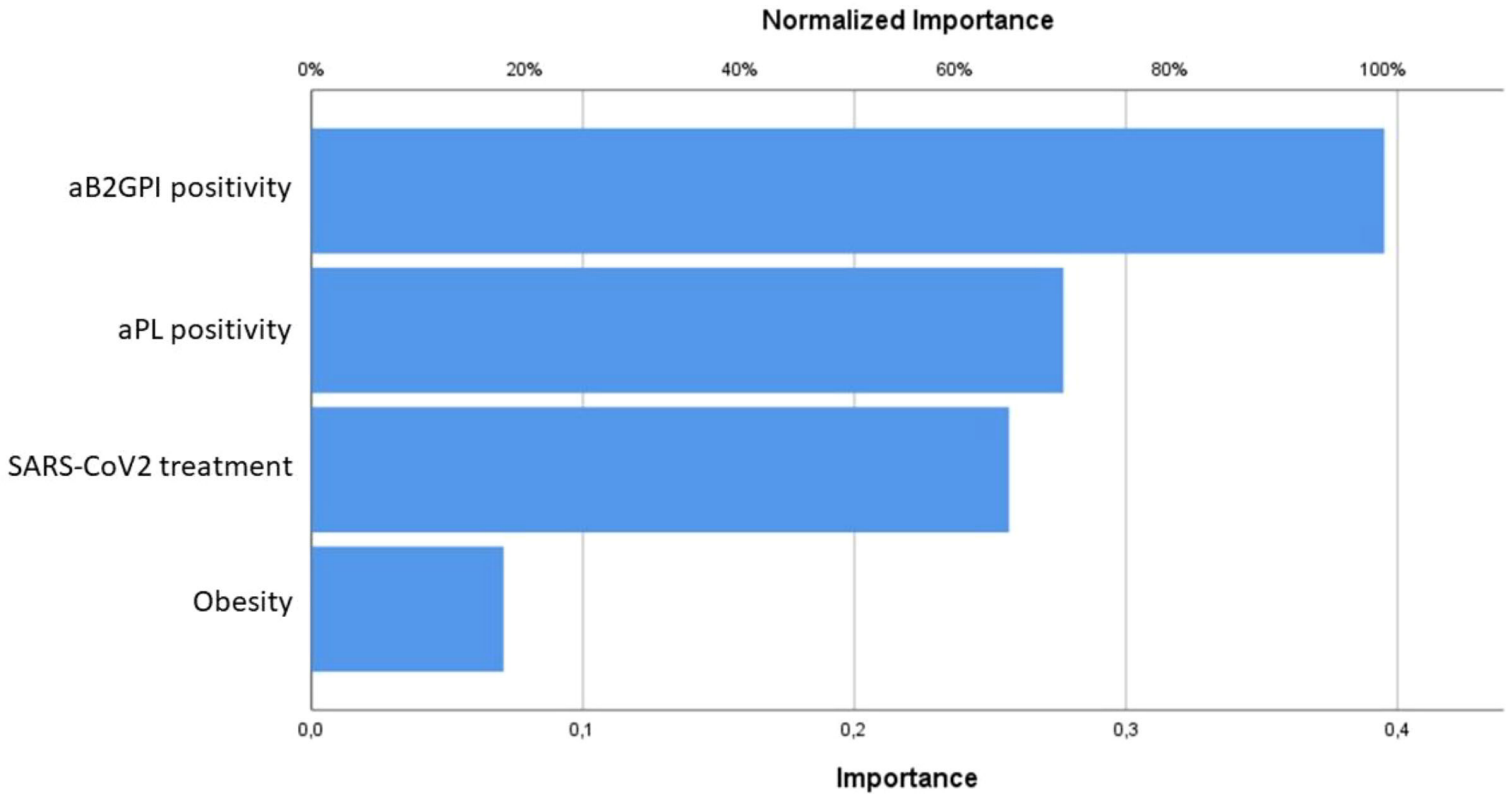
Predictive models resulting from the Multilayer Perceptron procedure for preeclampsia and HELLP syndrome, mainly influenced by aB2GPI and aPL positivity.

The statistical analysis about the obstetric complications associated with placental insufficiency due to aPL positivity was described in **Table 2**. **Table 3** collected the statistically significant results of the univariate logistic regression of confounding factors analyzed (only the significant ones shown): comorbidities (in particular obesity and hypothyroidism), SARS-CoV2 symptomatic infection and related treatment. Focusing on the objective of the study, the strongest inferential non-parametric significant association was found between HELLP syndrome and/or preeclampsia and positive aPL (p value = 0,004). Regarding the 5 patients with positive IgG and/or IgM aB2GPI (isolated or with positive aCL or LA) a statistically significant association with HELLP syndrome and/or preeclampsia (p value < 0,001) was confirmed. The multivariate logistic regression analysis did not confirm these associations; however, using the MLP procedure, we could verify the dependence of both preeclampsia and HELLP syndrome mainly on aB2GPI and aPL positivity (**Figure 1**).

**Table 2:** Pregnancy complications occurring in 151 patients, divided upon profile for antiphospholipid antibodies (aPL).

	Negative aPL (n=135)	Positive aPL (n=16)	P-value	Single positive aPL (n=12)	P-value	Double positive aPL (n=4)	P-value	aB2GPI positive (n=5)	P-value
Abruptio placentae	1/135(1%)	0	NS	0	NS	0	NS	0	NS
Gestational hypertension	5/135 (4%)	0	NS	0	NS	0	NS	0	NS
HELLP syndrome	0	1/16 (6%)	0,004	0	NS	1/4 (25%)	<0,001	1/5 (20%)	<0,001
ICU admission	3/135 (2%)	2/16 (13%)	0,030	1/12 (8%)	NS	1/4 (25%)	0,038	1/5 (20%)	0,034
IUGR	4/135 (3%)	1/16 (6%)	NS	0	NS	1/4 (25%)	NS	1/5 (20%)	0,034
LGA	0	1/16 (6%)	0,04	1/12 (8%)	NS	0	NS	0	NS
Maternal-fetal trasfusion	1/135 (1%)	0	NS	0	NS	0	NS	0	NS
Maternal thrombosis	1/135 (1%)	0	NS	0	NS	0	NS	0	NS
Neonatal complications	16/135 (12%)	3/16 (19%)	NS	1/12 (8%)	NS	2/4 (50%)	NS	0	NS
Oligohydramnios	3/135(2%)	0	NS	0	NS	0	NS	0	NS
PPH	10/135 (7%)	2/16 (13%)	NS	1/12 (8%)	NS	1/4 (25%)	NS	2/5 (40%)	0,007
Preeclampsia	7/135 (5%)	4/16 (25%)	0,004	1/12 (8%)	NS	3/4 (75%)	<0,001	3/5 (60%)	<0,001
pPROM	0	1/16 (6%)	0,004	1/12 (8%)	NS	0	NS	0	NS
SGA	4/135 (3%)	1/16 (6%)	NS	1/12 (8%)	NS	0	NS	1/5 (20%)	0,034
Threatened preterm delivery	2/135 (1%)	1/16 (6%)	NS	0	NS	1/4 (25%)	NS	1/5 (20%)	NS

aB2GPI, anti-beta2glycoprotein I; aPL, antiphospholipid antibodies; HELLP, hemolysis, elevated liver enzymes, and low platelets; ICU, Intensive care unit; IUGR, Intrauterine growth restriction; LGA, large for gestational age; NS, not significant; PPH, post-partum hemorrage; pPROM, preterm premature rupture of membranes; SGA, small for gestational age.

**Table 3:** Univariate logistic regression of statistically significant parameters and variables involved in pregnancy complications.

		aPL positivity	B2GPI positivity	Comorbidities	Obesity	Hypothyroidism	SARS-CoV2	SARS-CoV2 treatment
**Preeclampsia**	P-value	0,004	<0,001	0,005	–	–	–	0,003
	OR (95% CI)	6,1 (1,6-2,3)	25,9 (3,8-177,6)	5,5 (1,5-19,8)	–	–	–	8,1
**Abruptio placentae**	P-value	–	–	–	<0,001	–	–	(1,7-39,0)
	OR (95% CI)	–	–	–	1,2 (0,8-1,9)	–	–	–
**HELLP** **Syndrome**	P-value	0,004	<0,001	–	–	–	–	–
	OR (95% CI)	1,1 (0,9-1,2)	1,2 (0,8-1,9)	–	–	–	–	–
**ICU admission**	P-value	0,030	0,034	–	0,034	–	<0,001	–
	OR (95% CI)	6,3 (1,0-40,9)	8,9 (0,8-98,4)	–	8,9 (0,8-98,4)	–	1,1 (1,0-1,3)	0,005
**IUGR**	P-value	–	0,034	–	–	–	–	1,1
	OR (95% CI)	–	0,9 (0,8-98,4)	–	–	–	–	(1,0-1,2)
**LGA**	P-value	0,004	–	–	<0,001	–	–	0,005
	OR (95% CI)	1,1 (0,9-1,2)	–	–	1,3 (0,8-1,9)	–	–	1,1
**SGA**	P-value	–	0,034	–	–	–	–	(1,0-1,2)
	OR (95% CI)	–	8,9 (0,8-98,4)	–	–	–	–	–
**PPH**	P-value	–	0,007	–	–	–	–	–
	OR (95% CI)	–	9,1 (1,4-60,7)	–	–	–	–	–
**pPROM**	P-value	0,004	–	–	–	0,001	–	–
	OR (95% CI)	1,1 (0,9-1,2)	–	–	–	1,1 (0,9-1,3)	–	–
**Threat of preterm delivery**	P-value	–	0,003	–	–	–	–	–
	OR (95% CI)	–	18 (1,3-241,8)	–	–	–	–	–

aB2GPI, anti-beta2glycoprotein I; aPL, antiphospholipid antibodies; CI, confidence intervals; HELLP, hemolysis, elevated liver enzymes, and low platelets; ICU, Intensive care unit; IUGR, Intrauterine growth restriction; LGA, large for gestational age; OR, odds ratio; PPH, post-partum hemorrhage; pPROM, preterm premature rupture of membranes; SGA, small for gestational age.

Among the 16 women who tested positive for aPL, 7 (44%) had their aPL re-checked after at least 12 weeks: 3 became negative; 2 remained stable for both type and titre; 1 previously double positive patient turned out as a single positive; 1 single positive patient had a switch from positive LA to positive IgG aCL. **Table 4** shows descriptive data for each of the 16 aPL positive women.

**Table 4:** Description of the 16 patients who resulted positive for antiphospholipid antibodies (aPL).

	Age	Gestational age	Reason for hospitalization	Comorbidities	1st aPL test	Pneumonia	Obstetric complications	Neonatal complications	2nd aPL test
1	35y	39+2	Iterative caesarean section and tubal sterilization	ANA positivity	aB2GPI IgM+	No	Small for gestational age; Post-partum hemorrhage	No	aB2GPI IgM +
2	27y	41+3	Induction of labor	No	aCL IgG +	No	No	No	ND
3	28y	41+5	Induction of labor	No	LA +	No	No	No	ND
4	33y	10+4	SARS-CoV-2 bilateral interstitial pneumonia	Hypothyroidism	aCL IgG +	Yes	No	No	Negative
5	39y	38+3	Caesarean section	Behavioural disorders	aCL IgG + aB2GPI IgG+	No	Post-partum haemorrhage; Preeclampsia; HELLP syndrome	No	aCL IgG+ aB2GPI IgG -
6	28y	34+5	Suspected preeclampsia	No	aCL IgG + aB2GPI IgG+	No	Preeclampsia; Intrauterine growth restriction	Hypoglycaemia; Neonatal Intensive Care Unit admission	ND
7	27y	23+3	Urosepsis	Hypoacusis; Deficit XII factor	LA+ aB2GPI IgG+	No	Preeclampsia	No	Negative
8	19y	31	SARS-CoV-2 bilateral interstitial pneumonia	No	aCL IgG+ aB2GPI IgG+	Yes	Threat of preterm delivery	Respiratory distress syndrome	aCL IgG+ aB2GPI IgG +
9	35y	33	SARS-CoV-2 bilateral interstitial pneumonia; Caesarean section	ANA positivity; Hepatitis B	LA +	Yes	Preeclampsia	Respiratory distress syndrome; Neonatal necrotizing enterocolitis	ND
10	34y	40+6	Labour	No	aCL IgG+	No	No	No	ND
11	35y	13+6	Internal abortion	Multiple abortions	LA +	No	Internal abortion	No	ND
12	39y	18+3	SARS-CoV-2 bilateral interstitial pneumonia	Hypothyroidism, Multiple abortion	LA +	No	No	No	Negative
13	38y	25+5	Premature rupture of membranes	Hypothyroidism	LA +	No	Premature rupture of membranes	No	LA – aCL IgG + at high titre
14	28y	10+5	Hyperthyroidism	No	LA +	No	Hyperemesis	No	ND
15	32y	38+6	SARS-CoV-2 bilateral interstitial pneumonia	Behavioural disorders; Metachromatic leukodystrophy; Obesity	LA +	Yes	Urinary tract infection; Large for gestational age; Multiple placental infarctions	No	ND
16	40y	–	SARS-CoV-2 bilateral interstitial pneumonia	No	LA +	Yes	No	No	ND

aB2GPI, anti-beta2glycoprotein I; aCL, anti-cardiolipin; aPL, antiphospholipid antibodies; HELLP, hemolysis, elevated liver enzymes, and low platelets; LA, lupus anticoagulant; ND, No Data.

## Discussion

To the best of our knowledge, this is the first prospective study to investigate SARS-CoV-2 infection as a possible trigger of aPL positivity in pregnant women, with the aim of evaluating whether the overlap of these two conditions may result in an increased rate of obstetric and neonatal complications.

It is well recognized that infectious agents can play a dual role in the etiopathogenesis of APS, by acting as the initial trigger of the production of antibodies cross-reacting with aB2GPI and infectious peptides, and by inducing an inflammatory response. This is the so-called “two-hits theory” in which pathogenic aB2GPI act as the first hit and inflammatory response as the second one (13, 14). Moreover, several studies have shown that aCL occurred frequently during viral infections, particularly in HIV (49,7%), hepatitis B virus (HBV) (24%), and hepatitis C virus (HCV) (20%), while anti-B2GPI very rarely detected; aPL during the course of infections did not generally associate with thrombosis or other manifestations of APS (15).

The link between coagulopathy due to COVID-19 and positive aPL has been addressed in several studies, trying to define aPL as either an “epiphenomenon” or an active player in the pathogenesis (16). To shed light on this issue, Borghi et al. investigated the fine specificity of aPL detected in patients who were hospitalized for COVID-19-related thrombosis (17). A small group of patients (12,3%) were found to be positive for aPL at medium/low titre using chemiluminescence immunoassay, namely IgG/IgM/IgA aCL in 9,8/6,6/2,5% of patients and/or IgG/IgA/IgM aB2GPI in 5/5/0,8%. These aPL had different features from those found in patients with definite APS (reactivity against domain 1 and domain 4/5 of B2GPI was found in nearly 5% of the aPL positive cases) and there was no association between aPL positivity and thrombotic events. These aPL were mostly of the IgM isotype and at low titre, which are features classified under the definition of “low risk” aPL profile (12). Although this aPL profile is not strongly predictive of vascular events in APS, it is important to keep in mind that COVID-19 patients suffer from an acute form of systemic inflammation with complement activation (18), which may be responsible for endothelial perturbation. In this context, since aB2GPI can accumulate on the activated endothelium at high density, even low titers of aPL may become a pathogenic trigger for thrombosis. While transitory aPL are likely to be clinically non-significant in COVID-19 patients as in other infections (19), they may play a role in risk stratification of selected patients, with aPL being an additional risk factor acting synergistically with others. In this regard, we underline that only one case of maternal thrombosis was observed in our study, and it occurred in a patient with COVID-19 and negative aPL, confirming that COVID-19 itself can favor clots during a thrombophilic state like pregnancy.

Turning to obstetric APS, an aPL profile which is considered at “low risk” for thrombosis can actually mediate substantial damage, as high levels of B2GPI can be found in the placenta and bind aB2GPI antibodies, even at low titre (20). The presence of aPL was indeed found to be associated with an increased rate of obstetrical complications and fetal loss in the general obstetric population (21–24). In particular, it was demonstrated that the pathogenic mechanisms responsible for preeclampsia could be linked to a damage of the endothelium, ultimately leading to the release of vasoactive compounds (25) and that aB2GPI antibodies are able to promote endothelial activation (26). Therefore, Faden et al. assumed that the endothelial damage promoted by aB2GPI antibodies could be the starting point for the cascade of events leading to the preeclamptic syndrome and HELLP syndrome (27).

In November 2021, the US Centers for Disease Control and Prevention confirmed that pregnant women who develop COVID-19 have an increased risk of stillbirth, especially with the Delta type variant. Studies have also shown that placentas found positive for SARS-CoV-2 are typically characterized by a wide spectrum of pathological findings such as villous trophoblast necrosis, chronic histiocytic intervillositis and increased fibrin up to the level of massive perivillous massive deposition. These pathological lesions, in some cases together with placental hemorrhage, thrombohematomas and villitis, result in severe and diffuse placental parenchymal destruction (28). Before the pandemic, these lesions and subsequent placental insufficiency were typically associated with malperfusion conditions resulting from maternal diseases (maternal hypertension, diabetes mellitus, coagulopathies), fetal diseases (umbilical cord problems, abnormalities of placental development or placental implantation), environmental exposure to cigarette smoke, abruptio placentae, etc. Among the coagulopathies of autoimmune origin that are most associated with obstetric and neonatal complications there is indeed APS. Therefore, the pathogenic mechanisms underlying placental dysfunction and obstetric complications are complex, making it difficult to attribute adverse events to the direct viral effect, to the presence of aPL, or the combination of both factors.

Our study suggested that SARS-CoV-2 infection during pregnancy was not associated with the development of aPL. Recent studies reported the estimated prevalence of aPL positivity in the general population between 5 and 10%, in agreement with what we found in our patient cohort (11%) (24, 29–32). Moreover, we found a frequency of aB2GPI positivity similar to that described in a pioneer study that was conducted in our hospital by enrolling hundreds of pregnant women from the general obstetric population; we can assume that these figures from decades ago are still valid as the methods used for aPL detection have been validated for reproducibility overtime (27). Additionally, if we consider the total number of patients who presented with at least one obstetric complication, the frequency of positive aPL (28%) in this subgroup is similar to the one described in negative SARS-CoV-2 obstetric population (32–34).

In our cohort, obstetric complications occurred more frequently in aPL positive patients than in aPL negative ones. We might speculate that the interplay between aPL and SARS-CoV-2 infection could increase the risk of obstetric complications. By restricting the analysis to patients with positive aB2GPI, we found a significant association with complications mediated by placental dysfunction, namely preeclampsia and HELLP syndrome.

However, our study has limitations. We were not able to collect a control group of pregnant women with a negative swab test for SARS-CoV-2, due to the burden of multiple and severe wves of COVID-19 that hit our area. Moreover, the small number of aPL positive women and possible aPL-related manifestations limited the validity of statistical analysis. The heterogeneity of the cohort in terms of comorbidities, reasons for hospitalization, drugs intake, and gestational age also limited the interpretation of findings. For instance, the woman with confirmed positive aPL who developed HELLP syndrome had been taking antipsychotic medications, which are known to increase the chances for positive aPL (35).

In conclusion, our study showed that the frequency of positive aPL in pregnant women with SARS-CoV-2 infection is not greater than that described in the general obstetric population. Therefore, the infection itself does not seem to elicit an autoimmune response towards phospholipid-binding proteins like B2GPI. It might be possible that some women already carried aPL prior to the infection and the interaction between these two factors can facilitate the occurrence of obstetric complications, with particular reference to placental dysfunction leading to preeclampsia and HELLP syndrome. Based on our findings, aPL testing does not seem to be warranted in pregnant women with SARS-CoV-2 infection; however, aPL may contribute to risk stratification in women who present with concomitant risk factors and/or severe forms of COVID-19.

## Data availability statement

The raw data supporting the conclusions of this article will be made available by the authors, without undue reservation.

## Ethics statement

The studies involving human participants were reviewed and approved by Ethics Committee of Brescia (approval number NP4187). The patients/participants provided their written informed consent to participate in this study. Written informed consent was obtained from the individual(s) for the publication of any potentially identifiable images or data included in this article.

## Author contributions

LA, AT, FF, SZ, FP contributed to conception and design of the study. BN, MM, CZ, RO, SZ, FP, ES were involved in the enrollment of patients. MM, BN, GIG, EP, MGL, SP organized the database and performed the data collection. GIG, EP, CT performed the statistical analysis and prepared the tables. LA, GIG, EP wrote sections of the manuscript. All authors reviewed the manuscript and approved the submitted version.

## Conflict of interest

The authors declare that the research was conducted in the absence of any commercial or financial relationships that could be construed as a potential conflict of interest.

## Publisher’s note

All claims expressed in this article are solely those of the authors and do not necessarily represent those of their affiliated organizations, or those of the publisher, the editors and the reviewers. Any product that may be evaluated in this article, or claim that may be made by its manufacturer, is not guaranteed or endorsed by the publisher.
